# *N*-{4-Bromo-2-[(*S*)-menth­yloxy]-5-oxo-2,5-dihydro-3-fur­yl}-l-valine

**DOI:** 10.1107/S1600536809026129

**Published:** 2009-07-11

**Authors:** Xiu-Mei Song, Zhao-Yang Li, Zhao-Yang Wang, Jian-Hua Fu

**Affiliations:** a School of Chemistry and the Environment, South China Normal University, Guangzhou 510006, People’s Republic of China

## Abstract

The title compound, C_19_H_30_BrNO_5_, was obtained *via* a tandem asymmetric Michael addition–elimination reaction of 3,4-dibromo-5-[(*S*)-l-menth­yloxy]furan-2(5*H*)-one and l-valine in the presence of potassium hydroxide. The mol­ecular structure contains an approximately planar (r.m.s. deviation = 0.0204 Å) five-membered furan­one ring and a six-membered menth­yloxy ring adopting a chair conformation. The crystal packing is stabilized by inter­molecular O—H⋯O and N—H⋯O hydrogen bonding.

## Related literature

For applications of chiral 5-(l-menth­yloxy)-2(5*H*)-furan­ones, see: Feringa & De Jong (1988 ▶); De Koning *et al.* (1997 ▶); Lattmann *et al.* (1999 ▶); He *et al.* (2006 ▶); Wang *et al.* (2006 ▶). For biologically active 4-amino-2(5*H*)-furan­ones, see: Kimura *et al.* (2000 ▶); Tanoury *et al.* (2008 ▶). For related compounds, see: Wang *et al.* (2006 ▶); Li *et al.* (2009 ▶). For the synthesis, see: Chen & Geng (1993 ▶).
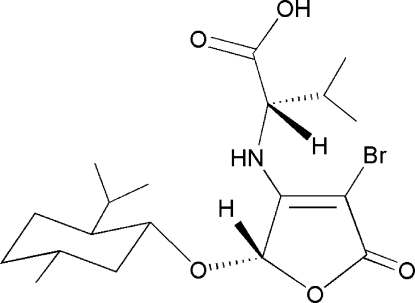


## Experimental

### 

#### Crystal data

C_19_H_30_BrNO_5_
*M* *_r_* = 432.34
Tetragonal, 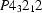

*a* = 10.5409 (9) Å
*c* = 39.388 (7) Å
*V* = 4376.4 (9) Å^3^
*Z* = 8
Mo *K*α radiation
μ = 1.91 mm^−1^
*T* = 293 K
0.30 × 0.22 × 0.18 mm

#### Data collection

Bruker APEXII area-detector diffractometer
Absorption correction: multi-scan (*SADABS*; Sheldrick, 2004 ▶) *T*_min_ = 0.558, *T*_max_ = 0.710
22304 measured reflections
3859 independent reflections
2726 reflections with *I* > 2σ(*I*)
*R*_int_ = 0.065

#### Refinement

*R*[*F* ^2^ > 2σ(*F* ^2^)] = 0.040
*wR*(*F* ^2^) = 0.085
*S* = 1.03
3859 reflections
241 parameters
H-atom parameters constrained
Δρ_max_ = 0.43 e Å^−3^
Δρ_min_ = −0.40 e Å^−3^
Absolute structure: Flack (1983 ▶), 1526 Friedel pairs
Flack parameter: −0.001 (11)

### 

Data collection: *APEX2* (Bruker, 2004 ▶); cell refinement: *SAINT* (Bruker, 2004 ▶); data reduction: *SAINT*; program(s) used to solve structure: *SHELXTL* (Sheldrick, 2008 ▶); program(s) used to refine structure: *SHELXTL*; molecular graphics: *SHELXTL*; software used to prepare material for publication: *SHELXTL*.

## Supplementary Material

Crystal structure: contains datablocks I, global. DOI: 10.1107/S1600536809026129/xu2542sup1.cif

Structure factors: contains datablocks I. DOI: 10.1107/S1600536809026129/xu2542Isup2.hkl

Additional supplementary materials:  crystallographic information; 3D view; checkCIF report

## Figures and Tables

**Table 1:** Hydrogen-bond geometry (Å, °)

*D*—H⋯*A*	*D*—H	H⋯*A*	*D*⋯*A*	*D*—H⋯*A*
N1—H1⋯O5^i^	0.86	2.28	3.047 (4)	149
O4—H4⋯O2^ii^	0.82	1.83	2.615 (3)	160

Symmetry codes: (i) 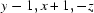; (ii) 
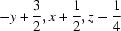
.

**Fig. 1.**
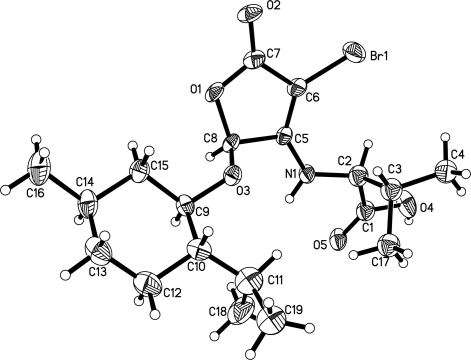
Displacement ellipsoid plot (30% probability level) of the title compound.

## supplementary crystallographic information

### Comment

Chiral 5-(*l*-menthyloxy)-2(5*H*)-furanones have been utilized as key building blocks in the synthesis of supramolecules and important natural products since 1980's (Feringa & De Jong, 1988; De Koning *et al.*, 1997; Lattmann *et al.*, 1999), especially in asymmetric synthesis (He *et al.*, 2006; Wang *et al.*, 2006). At the same time, 4-amino-2(5*H*)-furanone is an attractive moiety in chemical, pharmaceutical and agrochemical research. Many 4-amino-2(5*H*)-furanones have been patented as prodrugs or insecticides and herbicides (Kimura *et al.*, 2000; Tanoury *et al.*, 2008). Attracted by versatile 4-amino-2(5*H*)-furanones, we synthesized the title compound with chiral synthon 3,4-dibromo-5-(*S*)-(*l*-menthyloxy)-2(5*H*)-furanone and L-valine in the present of potassium hydroxide *via* the tandem asymmetric Michael addition-elimination reaction. With 2(5*H*)-furanone moiety and polyfunctional groups (carboxyl, amino, halo), the title compound is expected to be a biologically active product and excellent ligand.

The structure of the title compound is illustrated in Fig. 1. The title compound which has five chiral centers (C2(*S*), C8(*S*), C9(*R*), C10(*S*), C14(*R*)) contains a five-membered furanone ring and a six-membered menthyloxy ring connected each other *via* C8—O3—C9 ether bond. The furanone ring is approximately planar, whereas the cyclohexane ring displays a chair conformation with three substituents occupying equatorial positions. The bond lengths and angles in the title compound are good agreement with the expected values (Wang *et al.*, 2006; Li *et al.*,  2009). In the crystal structure the molecules are linked by intermolecular hydrogen bonds (Table 1).

### Experimental

The precursor 3,4-dibromo-5-(*S*)-(*l*-menthyloxy)-2(5*H*)-furanone was prepared according to the literature procedure (Chen *et al.*, 1993). An absolute ethanol solution (5 ml) of L-valine (4.5 mmol) and potassium hydroxide (5.8 mmol) was mixed with the dichloromethane solution (6 ml) of 3,4-dibromo-5-(*S*)-(*l*-menthyloxy)-2(5*H*)-furanone (3.0 mmol) under nitrogen atmosphere. The solution was stirred for 24 h at room temperature, and then the solvents were removed under reduced pressure. The solid residual was dissolved in dichloromethane, and pH of the solution was adjusted to 3 with 15% of aqueous HCl solution. Then the combined organic layers from extraction were concentrated under reduced pressure, and the crude product was purified by silica gel column chromatography with the gradient mixture of petroleum ether and ethyl acetate to give the product yielding (I) 0.9186 g (71.1%). Colorless crystals were obtained in acetone solution by slow evaporation.

### Refinement

The carboxyl H and imino H atoms were placed in calculated positions with O—H = 0.82 and N—H = 0.86 Å, and refined in riding mode with U_iso_(H) = 1.5U_eq_(O) and 1.2U_eq_(N). Methyl H atoms were placed in calculated positions with C—H = 0.96 Å and torsion angles were refined to fit the electron density, U_iso_(H) = 1.5U_eq_(C). Other H atoms were positioned in calculated positions with C—H = 0.97 (methylene) or 0.98 Å (methine), and were refined using a riding model with U_iso_(H) = 1.2 U_eq_(C).

### Crystal data

**Table:**

C_19_H_30_BrNO_5_	*D*_x_ = 1.312 Mg m^−^^3^
*M**_r_* = 432.34	Mo *K*α radiation, λ = 0.71073 Å
Tetragonal, *P*4_3_2_1_2	Cell parameters from 3973 reflections
Hall symbol: P 4nw 2abw	θ = 2.2–19.3°
*a* = 10.5409 (9) Å	µ = 1.91 mm^−^^1^
*c* = 39.388 (7) Å	*T* = 293 K
*V* = 4376.4 (9) Å^3^	Block, colourless
*Z* = 8	0.30 × 0.22 × 0.18 mm
*F*(000) = 1808.0	

### Data collection

**Table:**

Bruker APEXII area-detector diffractometer	3859 independent reflections
Radiation source: fine-focus sealed tube	2726 reflections with *I* > 2σ(*I*)
graphite	*R*_int_ = 0.065
φ and ω scans	θ_max_ = 25.0°, θ_min_ = 2.0°
Absorption correction: multi-scan (*SADABS*; Sheldrick, 2004)	*h* = −11→12
*T*_min_ = 0.558, *T*_max_ = 0.710	*k* = −12→9
22304 measured reflections	*l* = −40→46

### Refinement

**Table:**

Refinement on *F*^2^	Secondary atom site location: difference Fourier map
Least-squares matrix: full	Hydrogen site location: inferred from neighbouring sites
*R*[*F*^2^ > 2σ(*F*^2^)] = 0.040	H-atom parameters constrained
*wR*(*F*^2^) = 0.085	*w* = 1/[σ^2^(*F*_o_^2^) + (0.0221*P*)^2^ + 1.5196*P*] where *P* = (*F*_o_^2^ + 2*F*_c_^2^)/3
*S* = 1.03	(Δ/σ)_max_ = 0.001
3859 reflections	Δρ_max_ = 0.43 e Å^−^^3^
241 parameters	Δρ_min_ = −0.40 e Å^−^^3^
0 restraints	Absolute structure: Flack (1983), 1526 Friedel pairs
Primary atom site location: structure-invariant direct methods	Flack parameter: −0.001 (11)

### Special details

**Table:**

Experimental. Data for (I): [α]^20°^_D_ = 63.39° (c 0.437, CH_3_CH_2_OH); ^1^H NMR (400 MHz, CDCl_3_, TMS): 0.830 (3*H*, *d*, *J* = 6.8 Hz, CH_3_), 0.897–0.933 (7*H*, *m*, CH, 2CH_3_), 0.955–1.047 (8*H*, *m*, 2CH_3_, CH_2_), 1.316–1.451 (2*H*, *m*, 2CH), 1.610–1.708 (2*H*, *m*, CH_2_), 2.102–2.347 (3*H*, *m*, CH_2_, CH), 3.519–3.610 (1*H*, *m*, CH), 4.796 (1*H*, *s*, NH), 5.160–5.260 (1*H*, *m*, CH), 5.720 (1*H*, *s*, CH), 10.720 (1*H*, *s*, COOH); ESI-MS, *m*/*z* (%): Calcd for C_19_H_31_BrNO_5_^+^([*M*+H]^+^): 434.14, Found: 434.16 (95.0).
Geometry. All esds (except the esd in the dihedral angle between two l.s. planes) are estimated using the full covariance matrix. The cell esds are taken into account individually in the estimation of esds in distances, angles and torsion angles; correlations between esds in cell parameters are only used when they are defined by crystal symmetry. An approximate (isotropic) treatment of cell esds is used for estimating esds involving l.s. planes.
Refinement. Refinement of *F*^2^ against ALL reflections. The weighted *R*-factor *wR* and goodness of fit *S* are based on *F*^2^, conventional *R*-factors *R* are based on *F*, with *F* set to zero for negative *F*^2^. The threshold expression of *F*^2^ > σ(*F*^2^) is used only for calculating *R*-factors(gt) *etc*. and is not relevant to the choice of reflections for refinement. *R*-factors based on *F*^2^ are statistically about twice as large as those based on *F*, and *R*- factors based on ALL data will be even larger.

### Fractional atomic coordinates and isotropic or equivalent isotropic displacement parameters (Å^2^)

**Table:**

	*x*	*y*	*z*	*U*_iso_*/*U*_eq_	
Br1	0.60721 (4)	1.06205 (6)	0.066070 (11)	0.0784 (2)	
C1	0.3102 (4)	1.0150 (4)	−0.02299 (8)	0.0413 (9)	
C2	0.3657 (3)	0.9857 (3)	0.01146 (8)	0.0421 (9)	
H2	0.4503	1.0240	0.0126	0.051*	
C3	0.3802 (4)	0.8413 (4)	0.01711 (9)	0.0599 (12)	
H3	0.3900	0.8288	0.0416	0.072*	
C4	0.4988 (5)	0.7874 (5)	0.00061 (13)	0.105 (2)	
H4A	0.5138	0.7032	0.0090	0.157*	
H4B	0.5702	0.8404	0.0060	0.157*	
H4C	0.4875	0.7845	−0.0236	0.157*	
C5	0.3284 (3)	1.0733 (3)	0.06891 (8)	0.0367 (8)	
C6	0.4445 (3)	1.0829 (3)	0.08348 (8)	0.0447 (9)	
C7	0.4303 (4)	1.1255 (3)	0.11806 (8)	0.0457 (9)	
C8	0.2303 (3)	1.1000 (4)	0.09637 (7)	0.0398 (8)	
H8	0.1722	1.1677	0.0894	0.048*	
C9	0.0390 (3)	1.0016 (3)	0.11682 (8)	0.0410 (9)	
H9	−0.0103	1.0597	0.1026	0.049*	
C10	−0.0201 (4)	0.8704 (3)	0.11490 (10)	0.0522 (11)	
H10	0.0288	0.8163	0.1303	0.063*	
C11	−0.0114 (5)	0.8091 (4)	0.07965 (11)	0.0685 (13)	
H11	0.0778	0.8134	0.0728	0.082*	
C12	−0.1550 (4)	0.8779 (5)	0.12957 (12)	0.0746 (14)	
H12A	−0.2064	0.9322	0.1152	0.090*	
H12B	−0.1925	0.7939	0.1294	0.090*	
C13	−0.1566 (4)	0.9290 (5)	0.16525 (11)	0.0748 (14)	
H13A	−0.1137	0.8694	0.1801	0.090*	
H13B	−0.2438	0.9361	0.1728	0.090*	
C14	−0.0928 (4)	1.0580 (4)	0.16822 (9)	0.0602 (11)	
H14	−0.1425	1.1186	0.1548	0.072*	
C15	0.0400 (3)	1.0516 (4)	0.15277 (9)	0.0504 (10)	
H15A	0.0772	1.1358	0.1529	0.061*	
H15B	0.0929	0.9972	0.1667	0.061*	
C16	−0.0907 (5)	1.1053 (5)	0.20473 (10)	0.0854 (15)	
H16A	−0.1760	1.1192	0.2124	0.128*	
H16B	−0.0441	1.1835	0.2059	0.128*	
H16C	−0.0506	1.0431	0.2189	0.128*	
C17	0.2621 (5)	0.7689 (5)	0.00673 (12)	0.0897 (16)	
H17A	0.2539	0.7706	−0.0175	0.134*	
H17B	0.1889	0.8078	0.0169	0.134*	
H17C	0.2687	0.6826	0.0143	0.134*	
C18	−0.0868 (5)	0.8769 (5)	0.05239 (12)	0.0987 (18)	
H18A	−0.0734	0.8358	0.0309	0.148*	
H18B	−0.0595	0.9636	0.0510	0.148*	
H18C	−0.1754	0.8743	0.0580	0.148*	
C19	−0.0465 (6)	0.6681 (5)	0.08090 (16)	0.123 (2)	
H19A	−0.1369	0.6595	0.0827	0.185*	
H19B	−0.0070	0.6294	0.1003	0.185*	
H19C	−0.0175	0.6271	0.0606	0.185*	
N1	0.2878 (3)	1.0434 (3)	0.03785 (6)	0.0450 (7)	
H1	0.2098	1.0594	0.0330	0.054*	
O1	0.3055 (3)	1.1382 (2)	0.12568 (5)	0.0479 (7)	
O2	0.5122 (3)	1.1529 (3)	0.13854 (6)	0.0604 (8)	
O3	0.1660 (2)	0.9901 (2)	0.10285 (6)	0.0429 (6)	
O4	0.3943 (3)	0.9922 (4)	−0.04650 (6)	0.0843 (11)	
H4	0.3630	1.0057	−0.0652	0.127*	
O5	0.2048 (3)	1.0509 (3)	−0.02793 (6)	0.0596 (8)	

### Atomic displacement parameters (Å^2^)

**Table:**

	*U*^11^	*U*^22^	*U*^33^	*U*^12^	*U*^13^	*U*^23^
Br1	0.0465 (2)	0.1398 (5)	0.0488 (2)	0.0113 (3)	−0.0033 (2)	−0.0117 (3)
C1	0.047 (2)	0.051 (2)	0.0254 (19)	0.0110 (19)	−0.0007 (17)	−0.0051 (16)
C2	0.046 (2)	0.054 (3)	0.0255 (18)	0.0083 (18)	−0.0002 (15)	−0.0037 (16)
C3	0.093 (3)	0.058 (3)	0.029 (2)	0.023 (2)	−0.006 (2)	0.0020 (18)
C4	0.136 (5)	0.091 (4)	0.087 (4)	0.056 (4)	0.017 (4)	−0.007 (3)
C5	0.050 (2)	0.038 (2)	0.0221 (18)	0.0055 (18)	0.0006 (16)	0.0007 (15)
C6	0.052 (2)	0.057 (3)	0.0252 (17)	−0.002 (2)	−0.0005 (17)	0.0033 (16)
C7	0.061 (3)	0.050 (2)	0.0257 (18)	−0.010 (2)	−0.0028 (19)	0.0072 (17)
C8	0.051 (2)	0.044 (2)	0.0246 (18)	−0.001 (2)	0.0024 (16)	−0.0045 (18)
C9	0.042 (2)	0.047 (2)	0.034 (2)	0.004 (2)	0.0062 (17)	0.0082 (16)
C10	0.051 (3)	0.051 (3)	0.054 (2)	−0.004 (2)	−0.003 (2)	0.0120 (19)
C11	0.068 (3)	0.052 (3)	0.086 (4)	−0.004 (2)	−0.003 (3)	−0.018 (2)
C12	0.060 (3)	0.081 (3)	0.083 (4)	−0.016 (3)	0.007 (2)	0.014 (3)
C13	0.064 (3)	0.098 (4)	0.062 (3)	−0.004 (3)	0.018 (2)	0.019 (3)
C14	0.066 (3)	0.072 (3)	0.043 (2)	0.013 (3)	0.014 (2)	0.014 (2)
C15	0.058 (2)	0.058 (2)	0.035 (2)	0.000 (2)	0.0067 (18)	0.0044 (18)
C16	0.108 (4)	0.099 (4)	0.049 (3)	0.016 (4)	0.030 (3)	0.006 (3)
C17	0.132 (5)	0.068 (3)	0.070 (4)	−0.016 (3)	−0.010 (3)	−0.004 (3)
C18	0.121 (5)	0.114 (4)	0.061 (3)	0.016 (4)	−0.021 (3)	−0.026 (3)
C19	0.129 (5)	0.064 (3)	0.177 (6)	−0.020 (4)	−0.007 (4)	−0.038 (4)
N1	0.0439 (17)	0.066 (2)	0.0249 (15)	0.0106 (16)	−0.0028 (13)	−0.0058 (15)
O1	0.0584 (18)	0.0629 (19)	0.0225 (12)	−0.0081 (13)	0.0048 (12)	−0.0114 (12)
O2	0.070 (2)	0.082 (2)	0.0288 (14)	−0.0203 (15)	−0.0116 (14)	−0.0017 (13)
O3	0.0499 (15)	0.0424 (16)	0.0364 (14)	−0.0019 (13)	0.0063 (12)	−0.0002 (11)
O4	0.0685 (19)	0.158 (3)	0.0264 (14)	0.041 (2)	0.0074 (14)	0.0028 (16)
O5	0.0627 (19)	0.083 (2)	0.0334 (15)	0.0299 (17)	−0.0063 (12)	−0.0061 (14)

### Geometric parameters (Å, °)

**Table:**

Br1—C6	1.861 (4)	C11—C18	1.515 (6)
C1—O5	1.190 (4)	C11—C19	1.532 (6)
C1—O4	1.304 (4)	C11—H11	0.9800
C1—C2	1.509 (4)	C12—C13	1.505 (6)
C2—N1	1.458 (4)	C12—H12A	0.9700
C2—C3	1.546 (5)	C12—H12B	0.9700
C2—H2	0.9800	C13—C14	1.522 (6)
C3—C17	1.516 (6)	C13—H13A	0.9700
C3—C4	1.519 (6)	C13—H13B	0.9700
C3—H3	0.9800	C14—C16	1.522 (5)
C4—H4A	0.9600	C14—C15	1.528 (5)
C4—H4B	0.9600	C14—H14	0.9800
C4—H4C	0.9600	C15—H15A	0.9700
C5—N1	1.334 (4)	C15—H15B	0.9700
C5—C6	1.355 (5)	C16—H16A	0.9600
C5—C8	1.523 (4)	C16—H16B	0.9600
C6—C7	1.442 (4)	C16—H16C	0.9600
C7—O2	1.217 (4)	C17—H17A	0.9600
C7—O1	1.356 (4)	C17—H17B	0.9600
C8—O3	1.366 (4)	C17—H17C	0.9600
C8—O1	1.457 (4)	C18—H18A	0.9600
C8—H8	0.9800	C18—H18B	0.9600
C9—O3	1.452 (4)	C18—H18C	0.9600
C9—C15	1.511 (5)	C19—H19A	0.9600
C9—C10	1.518 (5)	C19—H19B	0.9600
C9—H9	0.9800	C19—H19C	0.9600
C10—C11	1.535 (5)	N1—H1	0.8600
C10—C12	1.537 (6)	O4—H4	0.8200
C10—H10	0.9800		
			
O5—C1—O4	125.2 (3)	C13—C12—C10	112.3 (4)
O5—C1—C2	125.0 (3)	C13—C12—H12A	109.1
O4—C1—C2	109.7 (3)	C10—C12—H12A	109.1
N1—C2—C1	109.7 (3)	C13—C12—H12B	109.1
N1—C2—C3	111.4 (3)	C10—C12—H12B	109.1
C1—C2—C3	111.7 (3)	H12A—C12—H12B	107.9
N1—C2—H2	108.0	C12—C13—C14	112.7 (3)
C1—C2—H2	108.0	C12—C13—H13A	109.0
C3—C2—H2	108.0	C14—C13—H13A	109.0
C17—C3—C4	111.9 (4)	C12—C13—H13B	109.0
C17—C3—C2	112.0 (4)	C14—C13—H13B	109.0
C4—C3—C2	112.8 (4)	H13A—C13—H13B	107.8
C17—C3—H3	106.5	C13—C14—C16	111.8 (3)
C4—C3—H3	106.5	C13—C14—C15	109.5 (3)
C2—C3—H3	106.5	C16—C14—C15	112.2 (4)
C3—C4—H4A	109.5	C13—C14—H14	107.7
C3—C4—H4B	109.5	C16—C14—H14	107.7
H4A—C4—H4B	109.5	C15—C14—H14	107.7
C3—C4—H4C	109.5	C9—C15—C14	112.5 (3)
H4A—C4—H4C	109.5	C9—C15—H15A	109.1
H4B—C4—H4C	109.5	C14—C15—H15A	109.1
N1—C5—C6	134.1 (3)	C9—C15—H15B	109.1
N1—C5—C8	118.5 (3)	C14—C15—H15B	109.1
C6—C5—C8	107.4 (3)	H15A—C15—H15B	107.8
C5—C6—C7	109.3 (3)	C14—C16—H16A	109.5
C5—C6—Br1	131.9 (2)	C14—C16—H16B	109.5
C7—C6—Br1	118.7 (3)	H16A—C16—H16B	109.5
O2—C7—O1	121.2 (3)	C14—C16—H16C	109.5
O2—C7—C6	128.8 (4)	H16A—C16—H16C	109.5
O1—C7—C6	109.9 (3)	H16B—C16—H16C	109.5
O3—C8—O1	110.9 (3)	C3—C17—H17A	109.5
O3—C8—C5	108.3 (3)	C3—C17—H17B	109.5
O1—C8—C5	104.1 (3)	H17A—C17—H17B	109.5
O3—C8—H8	111.1	C3—C17—H17C	109.5
O1—C8—H8	111.1	H17A—C17—H17C	109.5
C5—C8—H8	111.1	H17B—C17—H17C	109.5
O3—C9—C15	112.2 (3)	C11—C18—H18A	109.5
O3—C9—C10	106.5 (3)	C11—C18—H18B	109.5
C15—C9—C10	111.6 (3)	H18A—C18—H18B	109.5
O3—C9—H9	108.9	C11—C18—H18C	109.5
C15—C9—H9	108.9	H18A—C18—H18C	109.5
C10—C9—H9	108.9	H18B—C18—H18C	109.5
C9—C10—C11	113.9 (3)	C11—C19—H19A	109.5
C9—C10—C12	108.3 (3)	C11—C19—H19B	109.5
C11—C10—C12	114.6 (4)	H19A—C19—H19B	109.5
C9—C10—H10	106.5	C11—C19—H19C	109.5
C11—C10—H10	106.5	H19A—C19—H19C	109.5
C12—C10—H10	106.5	H19B—C19—H19C	109.5
C18—C11—C19	110.7 (4)	C5—N1—C2	124.9 (3)
C18—C11—C10	114.3 (4)	C5—N1—H1	117.6
C19—C11—C10	111.4 (4)	C2—N1—H1	117.6
C18—C11—H11	106.6	C7—O1—C8	108.9 (2)
C19—C11—H11	106.6	C8—O3—C9	117.2 (2)
C10—C11—H11	106.6	C1—O4—H4	109.5
			
O5—C1—C2—N1	−17.4 (5)	C12—C10—C11—C18	60.0 (5)
O4—C1—C2—N1	164.1 (3)	C9—C10—C11—C19	168.0 (4)
O5—C1—C2—C3	106.6 (4)	C12—C10—C11—C19	−66.5 (5)
O4—C1—C2—C3	−71.9 (4)	C9—C10—C12—C13	−56.3 (5)
N1—C2—C3—C17	76.8 (4)	C11—C10—C12—C13	175.3 (4)
C1—C2—C3—C17	−46.2 (4)	C10—C12—C13—C14	55.7 (5)
N1—C2—C3—C4	−155.9 (3)	C12—C13—C14—C16	−177.6 (4)
C1—C2—C3—C4	81.1 (4)	C12—C13—C14—C15	−52.6 (5)
N1—C5—C6—C7	177.2 (4)	O3—C9—C15—C14	−177.3 (3)
C8—C5—C6—C7	−5.1 (4)	C10—C9—C15—C14	−57.9 (4)
N1—C5—C6—Br1	1.8 (6)	C13—C14—C15—C9	53.7 (4)
C8—C5—C6—Br1	179.5 (3)	C16—C14—C15—C9	178.5 (3)
C5—C6—C7—O2	−174.9 (4)	C6—C5—N1—C2	13.4 (6)
Br1—C6—C7—O2	1.2 (5)	C8—C5—N1—C2	−164.1 (3)
C5—C6—C7—O1	2.2 (4)	C1—C2—N1—C5	−156.4 (3)
Br1—C6—C7—O1	178.3 (2)	C3—C2—N1—C5	79.5 (4)
N1—C5—C8—O3	66.1 (4)	O2—C7—O1—C8	179.3 (3)
C6—C5—C8—O3	−112.0 (3)	C6—C7—O1—C8	1.9 (4)
N1—C5—C8—O1	−175.8 (3)	O3—C8—O1—C7	111.4 (3)
C6—C5—C8—O1	6.1 (4)	C5—C8—O1—C7	−4.8 (4)
O3—C9—C10—C11	−51.4 (4)	O1—C8—O3—C9	91.0 (3)
C15—C9—C10—C11	−174.1 (3)	C5—C8—O3—C9	−155.3 (3)
O3—C9—C10—C12	179.8 (3)	C15—C9—O3—C8	−68.8 (3)
C15—C9—C10—C12	57.1 (4)	C10—C9—O3—C8	168.9 (3)
C9—C10—C11—C18	−65.5 (5)		

### Hydrogen-bond geometry (Å, °)

**Table:**

*D*—H···*A*	*D*—H	H···*A*	*D*···*A*	*D*—H···*A*
N1—H1···O5^i^	0.86	2.28	3.047 (4)	149
O4—H4···O2^ii^	0.82	1.83	2.615 (3)	160

Symmetry codes: (i) *y*−1, *x*+1, −*z*; (ii) −*y*+3/2, *x*+1/2, *z*−1/4.
